# Medical Progress

**Published:** 1894-02-10

**Authors:** 


### Medical Progress.

### DISEASES OF THE LUNG.

*Bronchial Catarrh.*—The value of TERPENE HYDRATE in the treatment of affections of the bronchial and nasal mucous membranes has been demonstrated by Dr. William Murrell[1], who once more directs attention to this remedy, and we quote his remarks *in extenso.* Its properties have been well known for many years, but in this country it has never been a popular remedy, and its claims seem to have been overlooked in favour of pure terebene and other similar compounds. It is a hydrate of turpentine, and is made by treating oil of turpentine with nitric acid and alcohol. It is a solid, and has somewhat the appearance of chloral hydrate. Its odour, which is slight, resembles that of pure terebene. The great difficulty in the way of its administration is that it is practically insoluble in water. It is usually said to dissolve in alcohol in the proportion of 1 in 10, but many specimens are far less soluble. On the Continent, where it enjoys a high reputation in the treatment of bronchial affections, it is used as a popular remedy in the form of an elixir. For some months past Dr. Murrell has prescribed it in a solution containing five grains to the half ounce, made up with simple elixir, and flavoured either with Virginian prune and syrup of tar, or with the aqua laurocerasi, or it may be made up with saccharine. Terpene not only relieves coughs and lessens bronchial secretion, but is a diuretic, and has been used with advantage in neuralgia.

*Acute Bronchitis.*—From laboratory experiments, and after a considerable clinical experience, Dr. R. W. Wilcox[2] highly recommends clorilana, the bark of the Cocillana Rusbyi, a plant indigenous to South America. The preparations he has used has been the fluid extract in doses of from five to twenty-five minims every three to six hours. He thinks the fluid extract preferable in many cases to a tincture, because it contains no alcohol. He has also used a syrup which contains all the active principles of the bark excepting those which are removed by precipitation by water from an alcoholic solution. The dose of the syrup is from one to two fluid drachms every four to six hours. Large doses produce nausea, dryness of the throat, and a desire to defæcate.

The glands and vessels are stimulated by the drug, especially those of the mucous membranes of the bronchial tubes and of the digestive system, with increase of secretion lasting some hours. Dr. Wilcox states that a hyperæmia of mucous membrane unaccompanied by secretion would be an indication for its use, and that the cases for which this remedy is useful are acute bronchitis, sub-acute and chronic, dry bronchitis, and chronic disease of pulmonary tissue. In these classes of cases he prefers cocillana to either ipecacuanha or morphine, and many other expectorants. Thus with cocillana (1) the expectorant effect can be obtained without risk of emesis, which is only induced by the injudicious exhibition of the drug; (2)

it requires from three to six hours in which to act, but its effect persists for the same period, and thus, once its action is established, the dose need not be so frequent as with ape morphine; (3) it increases the appetite, and its slight aperient action is beneficial.

"In cases of senile bronchitis it is contra-indicated, for it may add to the bronchorrhœa, so that it may become dangerous."

Bichromate of Potash is advocated as an expectorant by Dr. John Weaver[3]. "It is especially useful in both laryngitis and bronchitis if the secretion is tough and stringy and hard to raise." He values this remedy most highly in acute lacunar tonsilitis. He states that in treating laryngitis and bronchitis one must be careful not to use it too strong—not more than one grain well triturated to three or four ounces of water, a teaspoonful every half to two hours, as recommended by Dr. Hunt[4]. But in tonsilitis he employs it finely powdered, added to water until the latter is of a dark lemon or light orange shade, and of this a teaspoonful should be given every hour. In these cases the only limit to the administration is nausea. If this symptom appears he lessens the dose, and if necessary the frequency. "Tell your patient he will begin to improve by the third dose, and he will. But do not try weak solutions, or powders, or tablet triturates for the tonsilitis, or you may get disappointed."

[This remedy must be given cautiously, the dose not to exceed one-tenth grain, every six hours, in our opinion.—Ed.]

*Bronchiectasis.*—The value of intra-laryngeal injections of guaiacol and menthol in fœtid conditions of the sputa, lately reported by Dr. Grainger Stewart[5], has led Dr. J. McNaught[6] to report a case which well exemplifies its utility. The patient was an old woman, aged about sixty, subject to chronic bronchitis, in whom an acute complication arose, believed by Dr. McNaught to be abscess of the lung, for reasons specified. He remarks that "there is room for differences of opinion as to the diagnosis, but whatever the condition, the treatment of the fœtid expectoration by intra-laryngeal injections was most successful, not only in reducing temperature but improving the general state." After trying for a few days, without result, inhalation of iodine from a Robert's respirator inhaler, a solution consisting of guaiacol ʒiss., menthol ʒx., ol. olivæ ℥viii. was injected into the larynx, ♏xxx. being given at a time once a day, and three pills containing iodoform, creasoti aa. gr. i., croton chloral hydrate gr. ii. were given daily. After the use of two or three injections the fœtor almost ceased and the amount of expectoration was remarkably lessened, and in a fortnight was gone, and the purulent expectoration almost so. Simultaneously the temperature went down and the appetite improved. The patient was soon able to get up, and in five or six weeks she was convalescent from what seemed for a time to be a most threatening condition.

In another case of bronchitis, "where phthisis was suspected," he found intra-laryngeal injections produced most gratifying results.

*Hæmorrhagic Pleurisy.*—A case of hæmorrhagic pleurisy recently brought before the Medical Society of Hospitals by Dr. Hanot[7] (November 10th, 1893) led to an interesting discussion on the diagnostic import of sero-sanguineous exudation. In this case the attack of pleurisy was not so sudden in onset as usual. The pain in the side and difficulty in breathing only appeared a week after the date of exposure, and the patient had no rigor till the pain made its appearance. On aspiration blood-stained fluid was drawn off. By inoculation into the peritoneal cavity of a guinea-pig it was shown to be tubercular, although there was no evidence of tuberculosis in the patient, who left the hospital in three weeks apparently cured. Dr. Hanot drew attention to the fact that, as a general rule, in cases of hæmorrhagic pleurisy, premonitory to pulmonary tuberculosis, the effusion re-accumulates very rapidly after aspiration, and only disappears after a number of aspirations, although his case resembled those of Wintrich, supposed to be simple pleurises, in which the effusion of blood completely disappeared after a single aspiration. He therefore urges the importance of inoculation experiments in all such cases.

Dr. Netter had met with two cases of hæmorrhagic pleurisy, and in both inoculation demonstrated their tubercular nature, but he could mention several cases in which the inoculation of tubercular sero-fibrinous effusion gave negative results. Dr. Fernet remarked that although it is generally believed that hæmorrhagic pleurisy is in all cases dependent on tubercular changes, there could be no doubt it might occur independently of tuberculosis and cancer—*e.g.*, in Bright's disease.

*Pulmonary Actinomycosis.*—A case of actinomycosis reported by Dr. Netter[8] merits a careful note not only on account of the rarity of the affection, but more especially because the patient was cured by iodide of potassium. On September 13th an incision was made in the centre of an œdematous area on the left side of the thorax where fluctuation was detected. A small quantity of pus escaped, and continued to discharge. On September 16th the characteristic parasite was discovered, so there could be no doubt as to the diagnosis. The infection had probably originated in the mediastinum, whence the fungus had probably invaded the cellular tissue in front of the vertebral column, extending laterally, involving the parietal pleura until it appeared at the surface. Dr. Netter had recourse to the administration of iodide of potassium, which has proved so efficacious in bovine actinomycosis, commencing on October 5th with forty-five grains, the dose being doubled the following day, and continued in doses varying from thirty to seventy grains daily. Under its influence the general health of the patient rapidly improved, the induration around the sinus in the thoracic wall gradually disappeared. The fistula ceased discharging on the 26th, and a few days later it had been completely cured.

He mentions that there are only twelve recorded French cases. All came from the North of France or from Savoy, and in both regions animal actinomycosis is comparatively common. Borstrom has demonstrated that the fungus grows on the surface of certain graminaceæ, furnishing a common source of infection in man and beast. Thomassen is to be credited with the discovery of the efficacy of iodide of potassium in the treatment of "wooden tongue" in animals, and this remedy has since been used in actinomycosis in man and animals. Professor Nocard has since reported to Dr. Netter four cases of human actinomycosis he had previously treated with iodide of potassium, in all resulting in a cure. Local microbicidal treatment has been tried and failed.

*Emphysema.*—The further observations of Dr. A. G. Auld[9] on the morbid anatomy and pathology of emphysema render more complete his earlier communication (*Lancet*, July 2nd), in which he endeavoured to prove that the prevailing opinion as to the entirely atrophic character of the morbid alterations was only partially correct, inasmuch as productive and reactive phenomena in the air cells were the very earliest structural indications of impending emphysema. The earliest anatomical change consists in the enlargement and division of the nuclei in the walls of the air cells, a change Dr. Auld considers is reactive in character. He points to a possibly analagous change in the early stages of atheroma, wherein, owing presumably to increased tension in the vessel, the nuclei in the intima show proliferative changes. In old and advanced cases of emphysema this change often cannot be demonstrated, and it is doubtful whether in some at least it occurs, degenerative changes probably alone taking place, his observations leading him to believe that such degeneration is due to a nutritive alteration taking place, for the corpuscles of the cells in the air cells, and later the elastic fibres, show granular degeneration.

In the fibroid variety of emphysema, which Sir William Jenner regarded as the "consequence of the exudation of that variety of lymph which escapes from the capillaries when they are the seat of slight but long-continued congestion," Dr. Auld's observations support this view. But he directs attention to the fibroid alterations in cases of partial or localised emphysema, in which the fibroid changes are peculiar in their distribution, the outer coat of the pulmonary vessels, the inter-lobular septa, and regularly recurring groups of the pulmonary alveoli are invaded by a new and vascular tissue. This change appears to begin by the deposition here and there of small round clusters of nuclei, thickly set together. As throwing light on the occurrence of these degenerative changes Dr. Auld emphasises the associated occurrence of peculiar degeneration in the pulmonary nerves. In all cases of emphysema which he has examined, marked interstitial changes have been present in the nerves, with wasting and granularity of the fibres, even in those nerves not directly involved in the emphysema. He refers to the experiments of Brown-Séquard, which proved that irritation of the pneumogastric quickly develops emphysema, &c., from which he concluded that nervous disturbances in the pneumogastric play a prominent part in the production of emphysema. Dr. Auld considers that it is quite possible that a tropho-neurosis is engendered, due to the action of a poison in the nerve endings in the lung, *e.g.*, in whooping cough, and he cites, in this connection, the fact that emphysema may be due to gout. On the other hand, in those cases which seem dependent on chronic bronchitis, it may well be surmised that the chronic irritation which extends to the nerves in the fibrous tissues of the adventitia is such as to provoke the development of the disease and to render the action of the respiratory forces more speedy and certain. The rarity of emphysema in cases of hysterical cough he considers tends to confirm his view, the therapeutic import of which it is unnecessary to emphasise.

[1] Brit. Med. Jour., Vol. II., 1893. [2] Ther. Gaz., June 15, 1893. [3] New York Med. Rec., Sept., 1893. [4] Ibid., Aug. 12, 1893. [5] Brit. Med. Jour., June 3, 1893. [6] Ibid., June 24, 1893. [7] Med. Week., Nov. 17, 1893. [8] Ibid., Nov. 10. [9] Lancet, Dec. 2, 1893.

